# Foam Cells as Therapeutic Targets in Atherosclerosis with a Focus on the Regulatory Roles of Non-Coding RNAs

**DOI:** 10.3390/ijms22052529

**Published:** 2021-03-03

**Authors:** Amin Javadifar, Sahar Rastgoo, Maciej Banach, Tannaz Jamialahmadi, Thomas P. Johnston, Amirhossein Sahebkar

**Affiliations:** 1 Department of Allergy and Immunology, Mashhad University of Medical Sciences, Mashhad 9177948564, Iran; javadifarA971@mums.ac.ir (A.J.); rastgous971@mums.ac.ir (S.R.); 2 Department of Hypertension, Chair of Nephrology and Hypertension, Medical University of Lodz, 93338 Lodz, Poland; 3 Polish Mother’s Memorial Hospital Research Institute (PMMHRI), 93338 Lodz, Poland; 4 Department of Food Science and Technology, Quchan Branch, Islamic Azad University, Quchan 9479176135, Iran; jamiat931@gmail.com; 5 Department of Nutrition, Faculty of Medicine, Mashhad University of Medical Sciences, Mashhad 9177948564, Iran; 6 Division of Pharmacology and Pharmaceutical Sciences, School of Pharmacy, University of Missouri-Kansas City, Kansas City, MO 64108-2718, USA; Johnstont@umkc.edu; 7 Biotechnology Research Center, Pharmaceutical Technology Institute, Mashhad University of Medical Sciences, Mashhad 9177948564, Iran; 8 Applied Biomedical Research Center, Mashhad University of Medical Sciences, Mashhad 9177948564, Iran; 9 School of Pharmacy, Mashhad University of Medical Sciences, Mashhad 9177948954, Iran; 10 Department of Medical Biotechnology and Nanotechnology, School of Medicine, Mashhad University of Medical Sciences, Mashhad 9177948564, Iran

**Keywords:** atherosclerosis, lipid metabolism, noncoding RNAs, foam cell formation

## Abstract

Atherosclerosis is a major cause of human cardiovascular disease, which is the leading cause of mortality around the world. Various physiological and pathological processes are involved, including chronic inflammation, dysregulation of lipid metabolism, development of an environment characterized by oxidative stress and improper immune responses. Accordingly, the expansion of novel targets for the treatment of atherosclerosis is necessary. In this study, we focus on the role of foam cells in the development of atherosclerosis. The specific therapeutic goals associated with each stage in the formation of foam cells and the development of atherosclerosis will be considered. Processing and metabolism of cholesterol in the macrophage is one of the main steps in foam cell formation. Cholesterol processing involves lipid uptake, cholesterol esterification and cholesterol efflux, which ultimately leads to cholesterol equilibrium in the macrophage. Recently, many preclinical studies have appeared concerning the role of non-encoding RNAs in the formation of atherosclerotic lesions. Non-encoding RNAs, especially microRNAs, are considered regulators of lipid metabolism by affecting the expression of genes involved in the uptake (e.g., CD36 and LOX1) esterification (ACAT1) and efflux (ABCA1, ABCG1) of cholesterol. They are also able to regulate inflammatory pathways, produce cytokines and mediate foam cell apoptosis. We have reviewed important preclinical evidence of their therapeutic targeting in atherosclerosis, with a special focus on foam cell formation.

## 1. Introduction

Atherosclerosis is a chronic, progressive immuno-inflammatory disease that affects blood vessels and can result in the development of atherosclerotic plaques. These plaques have the potential to rupture and lead to cardiovascular disease, especially myocardial infarction (MI) or stroke, which represent two of the leading causes of death worldwide [1]. Since foam cells are a central player in the underlying pathology of atherosclerosis, reducing the formation of foam cells, or inducing their removal, might represent an effective therapeutic strategy. Recently, several groups reported that targeting foam cells could effectively ameliorate atherosclerosis, which are described in this review. Current studies have suggested that non-coding RNAs are involved in the pathophysiology and progression of atherosclerosis and are good diagnostic and prognostic biomarkers, as well as therapeutic targets [1]. In this review, we discuss non-coding RNAs as therapeutic targets in the context of either enhancing or attenuating the formation of foam cells, which ultimately affects the metabolism and overall homeostasis of cholesterol.

## 2. Pathophysiology of Atherosclerosis and the Key Role of Foam Cells

The pathophysiology of atherosclerosis includes a complex network of different cellular processes and is associated with risk factors such as arterial hypertension, smoking, hyperglycemia and hypercholesterolemia [1]. One of the triggers of this disease is endothelial damage, which plays an important role in the formation of atherosclerotic plaques. Endothelial damage results in arteries experiencing a decrease in nitric oxide (NO) bioavailability and an increase in the production of reactive oxygen species (ROS) [2,3]. NO is an anti-atherogenic and vasoprotective factor essential for vascular health and is obtained by conversion of arginine to citrulline by endothelial nitric oxide synthase III (ENOS) [4]. Increased ROS also produce a state of oxidative stress that assists in the modification of LDL to its oxidized form (oxLDL). Platelets can also be activated by oxLDL and induce vascular inflammation [5,6]. Eventually, oxLDL, together with chronic low-grade inflammation resulting from endothelial injury, trigger an innate immune response and increase the recruitment of immune cells, especially monocytes and neutrophils, to the subendothelial space to participate in plaque formation. Recruitment of monocytes to the subendothelial space is mediated by the upregulation of cell adhesion molecules and chemokines (such as monocyte chemoattractant protein-1 (MCP-1)) via oxLDL signaling pathways [7,8]. The recruited monocytes then differentiate into macrophages mediated by colony-stimulating cytokines such as M-CSF and GM-CSF, both of which are enhanced through oxLDL-induced signaling pathways [9]. Macrophages also differentiate into inflammatory (M1) macrophages in the presence of the Th1 cytokine, or anti-inflammatory (M2) macrophages in the presence of the Th2 cytokine, in the subendothelial environment [10,11]. No M1 and M2 macrophages have been identified as specific precursors for foam cell formation, but several studies have shown that M2 macrophages are more susceptible to foam cell formation [12].

Another aspect of the pathophysiology of atherosclerosis is the dysregulation of cholesterol metabolism in the macrophage, which is the main cell responsible for atherosclerotic plaque [13]. Oxidized-LDL signaling pathways are involved in the downregulation of cholesterol efflux transporters. By increasing both the internalization of oxLDL and the accumulation of lipid droplets in the macrophage, foam cell formation gradually occurs, which initially leads to fatty streaks and ultimately, to primary atherosclerotic lesions. Foam cells play a central role in the pathogenesis of atherosclerosis. Specifically, the formation and accumulation of foam cells in the subendothelial space of a damaged artery is one of the early key steps responsible for the development of atherosclerosis [14,15]. MCP-1 and TNF-α represent two inflammatory mediators released during foam cell formation. MCP-1 increases vascular smooth muscle cell (VSMC) proliferation and leukocyte migration and TNF-α upregulates cell adhesion molecules (CAMs), which subsequently increases the recruitment of VSMCs and immune cells [16]. The proliferation of VSMCs inside the plaque temporarily stabilizes the lesion through collagen synthesis and other extracellular matrix (ECM) proteins, which function to maintain the integrity of the fibrous cap and inhibit cap rupture. Nevertheless, an increasing number of foam cells, which have pro-atherogenic properties, ultimately leads to plaque rupture and blood vessel occlusion due to the release of matrix-degrading enzymes [17,18]. Most plaque growth, or lesion progression, is a result of macrophage activity, although other immune cells (e.g., neutrophils), have also been shown to play an important role [19]. Eventually, the plaque ruptures and results in clinical manifestations recognized as MI and stroke [20].

## 3. Foam Cells as Therapeutic Targets in Atherosclerosis

Foam cell formation is one of the critical processes in the development and pathogenesis of atherosclerosis. As mentioned above, fatty streaks are the first detectable “yellow “lesion in the vessel wall that indicates foam cell formation and the development of atherosclerosis. Foam cells are involved in the formation of primary atherosclerotic plaques, their continued growth and ultimately, their rupture, which finally, leads to either MI or stroke [5,12,14]. The specific therapeutic goals associated with each stage in the formation of foam cells and the development of atherosclerosis will be considered below. Processing and metabolism of cholesterol in the macrophage is one of the major steps in foam cell formation. Cholesterol processing involves lipid uptake, cholesterol esterification and cholesterol efflux, which ultimately leads to cholesterol equilibrium in the macrophage. However, dysregulation and disruption of these processes results in foam cell formation [21]. During the development of atherosclerosis, chemoattractants (especially MCP-1) recruit monocytes to the subendothelial space of the damaged endothelium and they undergo differentiation to macrophages [22]. The macrophages express scavenger receptors SRA-1, SRA-2, CD36 and LOX1. Cellular uptake of oxLDL occurs by phagocytosis and pinocytosis via scavenger receptors, which preferentially incorporate oxLDL relative to its native (unmodified/unoxidized) form [23].

Numerous studies in mice have selectively targeted either chemoattractants or adhesion molecules involved in the recruitment of monocytes, or cytokines involved in the transmigration of monocytes. This has been accomplished by inactivating the genes encoding various molecules, such as M-CSF, CCL2, CXCR1 and CCR5, which has been shown to result in less and smaller atherosclerotic lesions in ApoE^−/−^ mice [24,25,26]. Animal studies using LDLR^−/−^ mice have evaluated both the knockdown of VCAM-1 [27,28], or drug inhibition with a natural antioxidant AGI-1067, which showed successful and significant reductions in the development of atherosclerotic plaques in LDLR^−/−^ mice [29]. SRA-1, 2, CD36 and LOX-1 are the primary scavenger receptors of macrophages that play a key role in lipid uptake and are controlled by several regulators such as MEKK-2, MAP kinases and STAT1 [23,30,31,32]. Numerous studies have targeted the role of these three major scavenger receptors in foam cell formation. For example, overexpression of LOX-1 in ApoE^−/−^ mice accelerated the development of atherosclerosis, while LOX-1^−/−^, LDLR^−/−^ mice had smaller atherosclerotic lesions, which suggests a proatherogenic role for LOX-1 [33]. These receptors are multifunctional in nature and their expression is not limited to macrophages, because they are also expressed in aortic endothelial cells (ECs) and VSMCs. For these reasons, as well as the fact that different lipid uptake pathways exist, such as phagocytosis and pinocytosis, it is difficult to design studies that evaluate the therapeutic targeting of these receptors [34]. Additionally, an analysis of different studies indicates that there is still a debate between whether these receptors are pro-atherogenic, or anti-atherogenic [35,36]. Thus, we would suggest that further studies are needed to better understand the lipid uptake pathways and the true functional role of each of these receptors. In cholesterol esterification, ACAT-1 and neutral cholesterol ester hydrolase (NCEH) play a key role in catalyzing the esterification of cholesterol and removing cholesterol from macrophages, respectively [37,38]. ACAT-1 is found ubiquitously in the endoplasmic reticulum of cells and is sensitive to the degree of membrane cholesterol enrichment, which is why it functions to maintain the cholesterol content of cell membranes at an optimal level by catalyzing the esterification of excess free cholesterol [39]. Studies have shown that ACAT-1 gene knockout in ApoE^−/−^ and LDLR^−/−^ mice resulted in no change in the development of atherosclerotic lesions and failed to inhibit foam cell formation [40]. However, pharmacological inhibition of ACAT-1 in macrophages has been shown to increase foam cell formation in mouse and rabbit models of atherosclerosis [41,42]. Moreover, animal studies evaluating the suppression of NCEH1 and hormone-sensitive lipase (LIPE), two important enzymes involved in the hydrolysis of cholesterol esters (CEs) back to free cholesterol (FC), have demonstrated a significant increase in intracellular CE accumulation when compared to control animals [38,43]. This would suggest that future studies should target all three enzymes (NCEH1, LIPE and ACAT-1) to intentionally modulate cholesterol turnover in macrophages.

Cholesterol efflux is one of the primary events in cholesterol metabolism and foam cell formation. The mechanism for cholesterol efflux is mainly attributable to ATP-binding cassette transporters ABCA1, ABCG1, as well as SR-B1, which function to maintain cholesterol and phospholipid homeostasis in the macrophage [44]. ABCA1 facilitates cholesterol and phospholipid efflux and leads to the formation of ApoA1, while ABCG1 mediates cholesterol efflux to form nascent high-density lipoprotein (HDL), which ultimately prevents the formation of foam cells. The expression of these transporters is predominantly dependent on the activation of PPAR and LXRα transcription factors [45]. Many studies have targeted these specific transporters. For example, in LDLR^−/−^ mice treated with PPARα and PPARγ agonists, the progression of atherosclerosis decreased due to the increased expression of ABCG1 and ABCA1 [46,47].

One of the limitations of targeting these transporters involved in cholesterol efflux pathways is that other pathways, such as passive diffusion, exist to transport cholesterol throughout the cell [48]. In studies that have been conducted, including genetic ablation of both ABCA1 and SR-B1 simultaneously in ABCA1×SR-BI double knockout (dKO) mice, macrophage RCT was markedly impaired and macrophage foam cell formation was increased in both lung and Peyer’s patches of dKO mice. However, atherosclerotic lesions did not develop in these dKO mice, which was potentially due to the low levels of non-HDL-C [49]. It should be noted, however, that hepatic overexpression of ABCA1 in LDLr-KO mice leads to enhanced aortic atherosclerotic lesions [49]. Therefore, knockdown studies on these three transporters could best be described as having yielded mixed results [50].

Cholesterol efflux inhibits the accumulation of excess lipids in the foam cell. Over time, the ability of the foam cell to manage the extra lipoproteins in the foam cell decreases, which leads to endoplasmic reticulum stress and the production of ROS and, in turn, triggers the apoptotic cascade. Additionally, uncontrolled lipoprotein uptake itself causes foam cell apoptosis [51,52].

Eventually, apoptosis of foam cells leads to the release of pro-inflammatory cytokines such as IL-1, IL-6, TNF-alpha and matrix metalloproteinases (MMPs); all, of which, may aggravate atherosclerosis by promoting an inflammatory state and the infiltration of immune cells [53].

The use of either pharmacological anti-inflammatory agents such as canakinumab, adalimumab and the TNF inhibitor etanercept, or agents that attenuate oxidative stress, may hold promise as a therapeutic intervention [54,55].

Furthermore, trying to slow the death of foam cells might represent a therapeutic goal to prevent the development and worsening of atherosclerosis [56]. There are a number of strategies that could be employed to decelerate foam cell apoptosis. These include (1) targeting apoptotic pathways, such as the genetic inhibition of proapoptotic proteins such as BAX or Bcl-2 [57,58], (2) knockdown of apoptosis inhibitor of macrophages (AIM) [59], (3) targeting secondary necrosis pathways such as the clearance of apoptotic cells [60,61], (4) promoting efferocytosis of apoptotic macrophages by using LXR ligands or glucocorticoids [62,63], or (5) by activating PPARγ pathways [64]. Another strategy to either control foam cell formation or increase the clearance of foam cells is statin therapy. Statins have been extensively investigated in patients with cardiovascular disease due to their numerous pleiotropic effects such as reducing oxidative stress, enhancing plaque stability and exerting anti-inflammatory effects [65,66,67,68,69,70,71,72,73,74,75]. Following foam cell formation, the foam cells take on a profibrotic phenotype. By releasing substances such as MMPs, they increase monocyte recruitment and degradation of extracellular matrix proteins such as collagen and fibronectin. This process leads to plaque instability and ultimately, plaque rupture [53,76]. For this reason, inhibition of MMPs may represent a treatment option in atherosclerosis. Many studies have targeted MMPs, in particular MMP8, because its increase in human carotid plaque has been associated with an unstable plaque phenotype [77]. Using MMP8^−/−^ ApoE^−/−^ mice, it has been demonstrated that monocyte/leukocyte recruitment and macrophage lesions decrease. Moreover, either MMP-9 or MMP-12 deficiency in ApoE^−/−^ mice has been shown to increase plaque instability [78,79,80]. Historically, monocyte-derived macrophages were considered the major source of plaque foam cells. However, other cells in the arterial wall such as ECs and VSMCs, as well as stem/progenitor cells (SPCs), show a foam cell phenotype in atherosclerotic plaques [81,82]. In fact, studies have shown that 50% of the foam cell population originates from VSMCs in human atherosclerotic lesions [83]. Therefore, this may suggest yet another strategy in the treatment of atherosclerosis; namely, targeting alternative cellular origins of foam cells [84,85,86]. Finally, one of the most important strategies for targeting foam cell formation is the use of miRNAs, which will be discussed in detail in this article.

**Figure 1:**
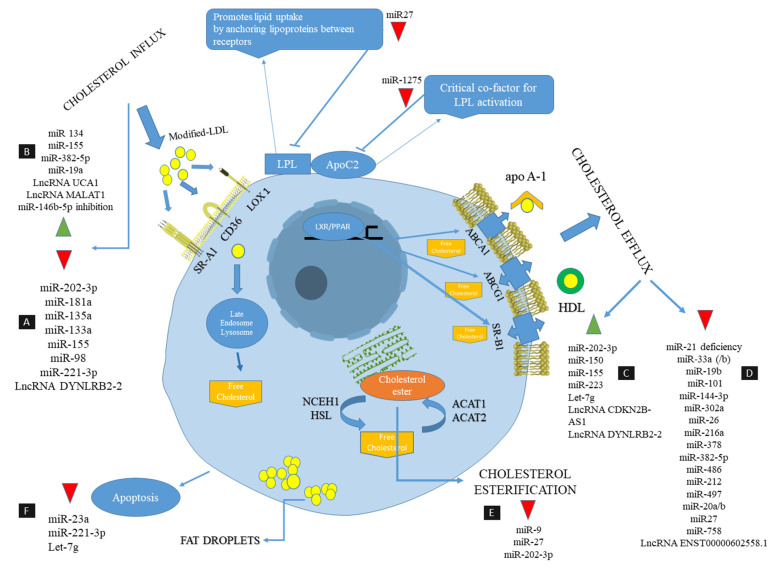
The mechanisms and molecules involved in cholesterol metabolism and homeostasis and the regulatory effects of Non-encoding RNAs are depicted in a foam cell. Cholesterol metabolism involves cholesterol influx, cholesterol esterification and cholesterol efflux, which ultimately leads to cholesterol homeostasis in the macrophage. Scavenger receptors SRA-1, CD36 and LOX1 are involved in oxLDL uptake. The gene expression of these receptors is downregulated in part A (see above); that is, non-encoding RNAs that have anti-atherosclerotic properties and are upregulated by part B ncRNAs, which are atherogenic and lead to lipid accumulation in macrophages. Cholesterol efflux is a pathway that transports excess free cholesterol from the cell, mainly via ABCA1, ABCG1, as well as SR-B1 transporters, leading to the formation of HDL and apoa1. The ncRNAs that reduce the formation of foam cells by increasing cholesterol efflux are specified in part C, as well as ncRNAs that lead to induction of the formation of foam cells by reducing cholesterol efflux, which are specified in part D. In cholesterol esterification, ACAT-1 and neutral cholesterol ester hydrolase (NCEH) play a key role in catalyzing the esterification of cholesterol and removing free cholesterol from foam cells, respectively. ACAT1 re-esterifies excess FC to promote the biosynthesis of CE that is stored in lipid droplets. In part E, microRNAs have been listed that reduce cholesterol esterification and increase the accumulation of free cholesterol, which ultimately promotes foam cell formation. Reducing foam cell apoptosis can stabilize atherosclerotic plaque and prevent the progression and worsening of atherosclerosis. ncRNAs that reduce foam cell apoptosis are shown in part F. 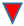 = decrease, 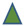 = increase.

The role and function of non-coding RNAs in foam cell formation and related processes (cholesterol efflux, cholesterol influx and cholesterol esterification) is depicted in Figure 1.

## 4. Non-Coding RNAs as Therapeutic Targets

The development of modern genomic and transcriptomic techniques, mass spectrometry and bioinformatics has led to a greater understanding concerning the role of non-coding RNAs (ncRNAs) in the regulation of gene expression involved in cellular and pathogenic disease processes, especially in atherosclerosis and the role of foam cells. Many studies have been conducted to identify the capacity of ncRNAs to alter or improve the progression of atherosclerosis at the foam cell stage [87,88,89,90]. ncRNAs are molecules that do not translate into proteins and the size of ncRNAs correlates with their function. There are different classes of ncRNAs in biological processes. For example, those involved in the regulation of gene expression, such as miRNAs, piRNAs and lncRNAs; those involved in the maturation of RNAs (snRNAs, snoRNAs); and those involved in the synthesis of proteins (rRNAs, tRNAs). The ncRNAs are typically classified by size. Thus, ncRNAs that are less than 200 nucleotides in length are categorized as small ncRNAs (sncRNAs), while those that are over 200 nucleotides in length are considered long ncRNAs (lncRNAs) [91,92]. The length of microRNAs is between 20–24 nucleotides, which bind to the 3’ untranslated region (3′UTR) of the target mRNA and either prevent its translation, or cause it to degrade. Most miRs reduce gene expression post-transcription, although in some instances, miRs can increase gene expression by activating the translation of the target mRNA. MiRNAs are often transcribed by RNA polymerase II (its early version was termed pri-miRNA) [93,94,95]. Biogenesis of miRNA occurs via the canonical pathway involving Drosha and Dicer, as well as through different non-canonical pathways that are independent of Drosha and even Dicer. Single mRNAs can be involved in different biological processes and can also be targeted by different miRNAs. Therefore, miRNAs play a multifunctional role due to the fact that they can participate in separate biological processes, which represents one of the limitations of using miRNAs in basic research and clinical investigations [96,97]. LncRNAs have a size between 200 bp to 100 kb, which are capable of binding to DNA, protein and RNA at various levels, exerting cellular regulation such as chromatin remodeling, mRNA splicing, or mRNA translation and multi-protein complex assembly. Additionally, lncRNAs are transcribed by RNA polymerase II and most of them undergo alternative splicing, 5′-capping and polyadenylation processes [98,99].

LncRNAs may function in both the *cis* and *trans* configuration to regulate the expression of target genes. They are also able to use a set of different molecular mechanisms to accomplish this goal. For example, they may act as a scaffold for the recruitment of chromatin modifiers or transcription factors, act as decoys for the breakdown of proteins and function as miRNA sponges to activate or deactivate target genes [100,101]. Presently, lncRNAs are classified by their genomic localization and modes of action or function, including intronic lncRNAs and intergenic lncRNAs that originate from a different region of the gene. Most mature transcribed lncRNAs are thought to have little potential for protein-coding, because some of them contain small open reading frames and encode small functional peptides. Interestingly, lncRNAs have more functional diversity due to the fact that they have less conserved sequences and are under less selective pressure [98,102,103].

In pathological conditions, therapeutic strategies involving miRNAs primarily use miRNA inhibition to reduce the expression of miRNAs whose expression has increased and use miRNA replacement to increase the expression of suppressed miRNAs.

miRNA inhibitors are chemically modified single-stranded antisense oligonucleotides (ASOs) that are complementary to the mature miRNA. To date, angtagomiRs (target-single miRNAs conjugated with cholesterol), anti-miRs (target-single miRNAs) and tiny anti-miRs (target miRNA families) have been synthesized to mediate miRNA inhibition. These ASOs can reduce pathogenic expressed miRNAs [91,92].

Nonviral delivery of miRNAs, for example, using liposomes, nanoparticles, or antibody-based methods, as well as viral delivery such as adeno-associated virus (AAV) and adenovirus have been used for the restoration of microRNA levels.

Since various studies have shown that ncRNAs have the potential to regulate different cell pathways, one promising therapeutic goal could be to manipulate their function using oligonucleotide inhibitors or miR mimics. Antisense oligonucleotides directed against specific miR sequences are effectively taken up by diverse tissues. Furthermore, miR mimics and inhibitors are relatively stable in plasma and miR mimics and inhibitors are not highly toxic and can easily reach cellular gene targets. However, the challenge to directly target a specific inflamed tissue and/or a specific cell line is still ongoing [87,88]. It should be noted that some studies using anti-miR have shown that they can target plaque macrophages and regulate gene expression in these cells [91,92].

Non-coding RNAs, which are involved in stimulating and reducing the formation of foam cells, are summarized in Table 1 and Table 2.

## 5. Non-Coding RNAs That Stimulate Foam Cell Formation/Function 

Most of the microRNAs described in this review, either directly or indirectly, target ABCA1. 

As mentioned above, ABCA1 is a membrane protein at the cell surface that regulates the transport of cholesterol and phospholipid and other metabolites [144]. In general, deficiency or downregulation of ABCA1 expression leads to a significant decrease in serum HDL levels and an increased risk of atherosclerosis. This is primarily due to the suppression of cholesterol efflux and disruption of the reverse cholesterol transport (RCT) cycle and subsequent foam cell formation. HDL has a cardioprotective role by preventing the oxidation of lipoproteins and returning cholesterol from peripheral tissues back to the liver via the RCT process [145].

Protein levels and ABCA1 activity are regulated at both the transcriptional and post-transcriptional levels, such that any downregulation of ABCA1 expression adversely affects the function of atheroprotective lipoprotein subclasses. Additionally, in APOE^−/−^ mice, the overexpression of ABCA1 can effectively reduce the size of atherosclerotic plaques [146,147].

**Figure 2:**
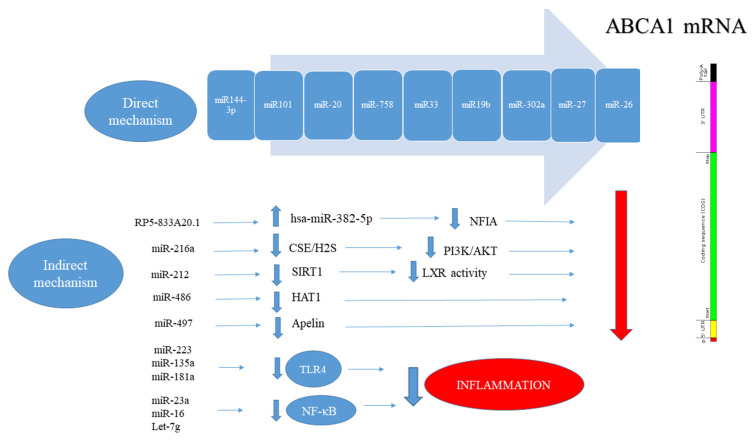
Direct and indirect effects of different Non-coding RNAs on the expression of ABCA1, which has a large 3′UTR region and is one of the most important transporters in cholesterol efflux [144,145]. In direct mechanism: non-coding RNAs able to bind to 3′UTR of ABCA1 mRNA transcript which ABCA1 expression regulate, in indirect mechanism: non-coding RNAs were not able to bind to 3′UTR of ABCA1 mRNA transcript although these noncoding RNAs that bind to 3′UTR of other mRNA transcript in turn regulate ABCA1 expression. It has also been shown that reducing inflammation increases the expression of this transporter and has a negative role in the formation of foam cells by increasing cholesterol efflux [148,149].

Direct and indirect effects of different Non-coding RNAs on the expression of ABCA1 are shown in Figure 2.

Some microRNA, such as miR-33 [104], miR-19b [106], miR-101 [106], miR-144-3p [106], miR-302a [104], miR-26 [106], miR-20a/b [106] and miR-758 [106] directly target the 3′UTR of ABCA1 in macrophages, which suppresses ABCA1 expression and disrupts cholesterol homeostasis. The disruption in cholesterol homeostasis occurs due to a decrease in the efflux of cholesterol to ApoA1 or HDL, which ultimately causes the formation of foam cells. A more detailed description is given in Table 1.

### 5.1. miR-33

This microRNA directly targets ABCA1 and ABCG1 in murine and human macrophages and downregulates these transporters, which leads to lower cellular cholesterol efflux and increased cholesterol accumulation and foam cell formation. In human cells, miR-33 neither downregulated the expression of ABCG1, nor interfered with cholesterol efflux to HDL in human THP-1 macrophages, which is consistent with the lack of miR-33-binding sites in the human ABCG1 3′UTR [150,151,152]. In a study using double-knockout miR-33^−/−^, ApoE^−/−^ mice, cholesterol efflux increased and plaque size decreased compared to control mice. Moreover, it was demonstrated that the use of microRNA-33 antagonism increased ABCA1 expression in vitro and in vivo and increased cholesterol efflux to ApoA1 [105,152,153]. In another study, the silencing of miR-33 with antisense oligonucleotides (anti-miR-33) in LDLR^−/−^ mice for 14 weeks did not affect lesion formation and the progression of atherosclerosis and failed to maintain elevated plasma HDL-cholesterol (HDL-C) [104]. This is probably because previous regression studies employed mice that received anti-miRs for only 4 weeks, as well as the existence of homeostatic compensatory mechanisms controlling plasma HDL. Additionally, the discrepancy between experimental designs, including the dietary composition, the gender of the mice and the possible influence of the intestinal microbiome may vary between the two models [154]. There is evidence suggesting athero-protective effects of hematopoietic loss of miR-33 including decreased number of apoptotic cells and decreased size of the necrotic cores in plaques from mice transplanted with miR-33^−/−^ bone marrow, indicative of enhanced plaque stability. Moreover, upregulation of MERTK experssion, a kinase involved in the regulation of efferocytosis, increases ABCA1 expression, MQs cholesterol efflux capacity and RCT and decreases the accumulation of cholesterol esters (CEs) in MQs in vivo. Furthermore, loss of miR-33 remarkably decreases the amount of MQ phosphatidylethanolamines (PEs) and phosphatidylserine (PS). These changes may have important effects such as attenuating NFKB activation and innate immune response [155].

Despite athero-protective effect of anti-miR-33 therapies, global deletion of miR-33 results in disturbed responsiveness to insulin in multiple metabolic organs including the liver, white adipose tissue (WAT) and skeletal muscle through TAGs, DAGs and ceramides accumulation, PKC activation and inhibition of ERK activity. Furthermore, alterations in feeding behavior are mainly responsible for the obesity in miR-33^−/−^ mice. Since the hypothalamic POMC and AgRP neurons, which regulate food intake, are also GABAergic neurons, they may be directly influenced by alterations in miR-33. This could elucidate some of the differences between genetic and pharmacological inhibition of miR-33, as antimiR-33 therapeutics would not be expected to pass the blood-brain barrier [156].

### 5.2. miR-27a/b

The miR-27 family has two isoforms; namely, miR-27a, which is an intergenic miRNA, and miR-27b, which is an intronic miRNA. miR-27a/b has been reported to regulate several genes involved in lipid metabolism, including PPARs and RXRs, which can activate the transcription of many target genes in vivo, including SR-BI, ABCG1 and ABCA1 [157]. This miRNA, by repressing CD36 expression in THP-1 macrophages, regulates cholesterol uptake. Additionally, miR-27a/b reduces the expression of ACAT1 (involved in the formation of foam cells via re-esterification of excess FC as a means to increase CEs), which, in turn, leads to a decrease in the formation of CEs in THP-1 macrophage-derived foam cells [157]. To maintain the homeostasis of cholesterol in macrophages, intracellular CE is hydrolyzed to FC as the initial step for the efflux of excess cholesterol, which occurs via the ABC transporters ABCA1 and ABCG1, as well as other non-transporter pathways such as SR-B1 [157]. Moreover, miR-27a/b inhibits LPL expression, which is involved in lipid uptake in vitro and in vivo. Importantly, this miRNA also regulates HDL-mediated cholesterol efflux in human foam cells without targeting SRBI and ABCG1, which probably indirectly affects the PPAR/RXR pathway. Additionally, miR-27a/b inhibits cholesterol efflux from macrophages via reduction in the expression of ABCA1 and subsequently causing a decrease in ApoA1 [110,158,159].

Some microRNAs, such as miR-216a [114,160], miR-486 [114,160], miR-212 [114,160], miR-497 [114,160] and LncRNA ENST00000602558.1 [118], also indirectly decrease the expression of the ABCA1 gene. The details are provided in Table 1.

### 5.3. miR-216a

The expression of this particular miRNA has been shown to be increased in myocardial biopsies from patients with ischemic heart failure [161].

It is known that the cystathionine gamma-lyase/hydrogen sulfide (CSE/H_2_S) enzymatic reaction in the trans-sulfuration pathway (a pathway that generates endogenous H_2_S) increases ABCA1 expression in foam cell [160,162,163,164].

Specifically, miR-216a directly targets the 3′UTR of CSE, which is one of the two key enzymes in endogenous H_2_S production. The CSE/H_2_S system has an anti-atherosclerotic role in the cardiovascular system. Downregulation of the CSE/H_2_S enzymatic reaction pathway results in decreased ABCA1 expression due to a reduction in the phosphorylation of PI3K and AKT, which subsequently leads to an increase in cholesterol accumulation in foam cells [114,160].

### 5.4. miR-382-5p

The RP5-833A20.1/miR-382-5p/NFIA pathway is essential for the regulation of cholesterol homeostasis and inflammatory responses. Interestingly, RP5-833A20.1 has been shown to decrease nuclear factor IA (NFIA) expression through the hsa-miR-382-5p pathway. NFIA is essential for adipocyte differentiation and lipid droplet formation. Specifically, NFIA inhibits atherosclerotic plaque formation in ApoE^−/−^ mice by increasing RCT and decreasing circulating cytokine levels. Overexpression of miR-382-5p causes a reduction in the expression of NFIA, as well as decreases the expression of ABCA1 and ABCG1 and increases the expression of SRA1, CD36 and NFKB [107,165]. In vivo studies with miR-382-5p have not been performed to date.

## 6. Non-Coding RNAs That Attenuate Foam Cell Formation

It has been shown that some non-coding RNAs are involved in macrophage cholesterol homeostasis by acting on inflammatory pathways. Studies have shown that miR-181a [126], miR-135a [126] and miR-223 [126], target TLR4 and reduce its expression. Thisprocess regulates cholesterol homeostasis in macrophages by reducing inflammation, reducing lipid uptake and increasing cholesterol efflux. Within macrophages, MiR-23a [126], miR-16 [126] and Let-7g [126] have an inhibitory effect on NF-kB pathways, which regulate lipid metabolism by reducing inflammation. The details are summarized in Table 2.

### 6.1. miR-150

MiR-150 has been shown to be upregulated in a human monocyte cell line in response to oxLDL treatment. Overexpression of this microRNA was proven to decrease the endogenous expression of AdipoR2 in macrophages, which promotes cholesterol efflux by the upregulation of ABCA1 and ABCG1. This process occurs via the PPARγ- and LXRα-dependent signaling pathways and decreases CD36 and intracellular lipid accumulation.

Since miR-150 can be packaged into microvesicles (MVs) and secreted from monocytes, MVs isolated from the plasma of patients with atherosclerosis have been determined to possess greater amounts of miR-150 than those from healthy donors. This finding may be explained by the fact that during the pathogenesis of atherosclerosis, stimulation of monocytes to release MVs that contain miRNAs, such as miR-150, can prevent lipid accumulation and foam cell formation [130].

### 6.2. miR-155

Dual effects of miR-155 on macrophages in the context of atherosclerosis.

miR-155 is a “multi-target” molecule specifically expressed in atherosclerotic plaques and pro-inflammatory macrophages [166]. The effects of miR-155 on atherogenesis have been controversial, because it exerts dual effects on both inflammatory and apoptotic pathways in lesional macrophages. miR-155 can enhance or prevent lesion formation relevant to the “stage” of atherosclerosis. Nazari-Jahantigh et al. reported that miR-155 was anti-atherogenic in the early stage of atherosclerosis, but became pro-atherogenic as the lesions progressed to a more advanced stage [167]. OxLDL induces miR-155 expression in human macrophages, which is essential for lipid uptake and ROS production by macrophages [168]. The use of antagomiR-155 has been shown to reduce lipid levels in human macrophages and decrease the size of plaques in ApoE^−/−^ mice. Elevated miR-155 levels increase oxLDL-induced foam cell formation by targeting HBP1. In addition, miR-155 expression is downregulated by the YY1/HDACs complex [113].

Another study showed that miR-155 mimics enhanced the expression of CEH at both the transcriptional and translational level in a dose- and time-dependent manner in human foam cells, although this effect may have occurred due to the inhibition in the expression of Tim-3, since overexpression of Tim-3 can inhibit the expression of CEH. Additionally, using a human monocyte cell line, it has been demonstrated that the overexpression of miR-155 can significantly inhibit the expression of SR-A, decrease lipid accumulation, increase the expression of ABCA1 and thereby increase cholesterol efflux [132].

A study also found that NF-κB transcription factor, which is activated by TNF-α, binds to the miR-155 promoter and stimulates transcription of miR-155. Accordingly, the expression of calcium-regulated heat-stable protein 1 (CARHSP1) is decreased. This is significant, because CARHSP1 enhances the stability of TNF-α mRNA, which is important for the efficient production of TNF-α. Furthermore, it has been demonstrated in a human monocyte cell line that miR-155 indirectly decreases TNF-α expression, decreases macrophage inflammation and lipid uptake and decreases foam cell formation [133].

## 7. Conclusions

Atherosclerosis is a chronic disease with a network of complex pathological processes. One of the primary mechanisms involved in this disease is the formation of foam cells, which leads to the growth and rupture of atherosclerotic plaques and finally, to its clinical manifestations (MI and stroke) [12,169]. Noncoding RNAs play an important role in various aspects of atherogenesis including foam cell formation. For example, many studies using noncoding RNAs have targeted different aspects of foam cell formation, such as cholesterol uptake, storage and efflux, indicating the well-established and effective role of non-coding RNAs, particularly miRNAs, in foam cell formation. Noncoding RNAs, especially miRNAs such, as miR-33, miR-27ab and miR-144, can have a pro-atherosclerotic role by stimulating foam cell formation, while others, such as miR-150, miR-1275 and Let-7g, exert an atheroprotective role by suppressing foam cell formation. miR-155 has shown conflicting results in different studies.

Clinical work performed using miRNAs has focused primarily on the inhibition of miRNAs through antisense oligonucleotides that complement targeting mRNA. For example, anti-miR oligonucleotides, modified antimiR oligonucleotides, anti-miR peptides, or the use of genetic vectors to replace miRs, such as with miR mimics, have been used [87,88,89,90]. Today, many preclinical studies have investigated the therapeutic anti-atherosclerotic potential of miRNAs by using miR mimics or their antagonists, which have shown promising results. These findings indicate that miRNA targeted therapy may represent a novel approach for the treatment of atherosclerosis. However, chronic treatment or genetic ablation of some of these miRNAs (miR-33), has been found to result in adverse effects including obesity and insulin resistance [156].

There are also limitations to using miRNAs, including their multifunctionality and their role in different biological processes. Moreover, there are few specific miRNAs in a tissue or organ, so most of the miRNAs affect unselected or non-targeted tissues. One of the major drawbacks to the therapeutic use of microRNAs in atherosclerosis is the fact that many molecules of this class have additional unwanted side effects and studies on the interaction between different miRNAs are relatively scarce. Secondly, miRNAs target prediction tools are not completely accurate and false positive and false negative predictions are still an issue of concern. Lastly, the full scope of elucidating their functions in vivo is limited and this fact impedes the investigation of the most interesting primate-specific non-coding RNAs in widely used atherosclerotic mouse models. Furthermore, the discrepancies in anatomy, lipid metabolism and gene expression complicate the translation of experimental results obtained in mice to humans.

## Figures and Tables

**Table 1:** Non-coding RNAs that stimulate foam cell formation/function.

Non-Coding RNAs	Expression Type	Target Gene	Genetic Information (Human)	Experimental Model		Effect on Foam Cell Formation	Regulation in Lipid Metabolism	Reference
				In Vivo	In Vitro			
**miR-144-3p**	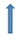	ABCA1	LXR and FXR control miR-144 expression, with both causing an upregulation in this miRNA	ApoE^−/−^ mice	human THP-1 macrophage-derived foam cells	(+) Decrease cholesterol efflux to both apoAI and HDL	decreased HDL-C circulation and impaired RCT in vivo 144-3p mimics (agomir) increases the expression of inflammatory factors such as IL-1b, IL-6 and TNF-α in vivo and in vitro	[103]
**miR-33(a/b)**	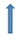	ABCA1, NPC1 ABCG1	encoded within intron 16 of SREBF2, a gene that encodes a key transcriptional regulator of cholesterol uptake and synthesis	miR-33^−/−^ ApoE^−/−^ mice	THP-1 macrophage-derived foam cells	(+) Decrease cholesterol efflux	miR-33 coordinates cholesterol homeostasis	[104,105]
**miR-19b**	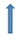	ABCA1	located on chromosome 13q31.3 By an unknown mechanism, expression was increased compared with the control group in advanced human atherosclerotic plaques obtained from patients during endarterectomy	ApoE^−/−^ mice	human THP-1 macrophage-derived foam cells	(+) Decrease the efflux of cholesterol to apoAI	Decrease the levels of HDL (inhibitory role in RCT)	[106]
**miR-101**	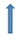	ABCA1	located on chromosome 1p31.3	-	human THP-1-derived macrophages	(+) Decrease the efflux of cholesterol under inflammatory conditions	regulates the availability of free cholesterol for cellular efflux by inhibiting autophagy	[107]
**miR-378**	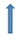	ABCG1	The level of miR-378 in the aorta is elevated during the progression of atherosclerosis in apoE^−/−^ mice	ApoE^−/−^ mice	ox-LDL-treated human THP-1 macrophages	(+) Decrease cholesterol efflux	activator protein-1/miRNA-378/ABCG1 is a novel cascade for CoQ10 in facilitating macrophage cholesterol efflux in vitro and in vivo	[108]
**miR-302a**	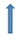	ABCA1	located on chromosome 4q25	Ldlr^−/−^ mice	primary human and murine macrophages	(+) Decreases efflux of cholesterol	inhibiting miR-302a in vivo increases ABCA1 in aorta and liver of Ldlr^−/−^ mice and increases circulating HDL	[109]
**miR-27**	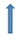	LPL, ACAT1, ABCA1 CD36	located on chromosome 9q22	-	ox-LDL-treated THP-1 macrophages	(+) Reduced cholesterol efflux reduces cholesteryl ester formation blocks lipid uptake	regulates the ratio of cellular free cholesterol (FC) and cholesterol ester (CE) in THP-1 macrophages	[110]
**miR-26**	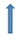	ARL7, ABCA1	located on chromosome 3p22.2	-	mouse and human LXR activated macrophages	(+) Downregulate LXR dependent cholesterol efflux	blocks the expression of two important LXR target genes (ABCA1 and ARL7) required for cholesterol efflux	[111]
**miR-134**	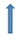	ANGPTL4	located on chromosome 14q32.31	-	THP-1 macrophages	(+) Increase LPL-mediated lipid accumulation	By suppressing the expression of ANGPTL4, which is a regulator of lipoprotein lipase (LPL) activity, it promotes oxLDL uptake and inflammatory responses in vitro	[112]
**miR-155**	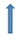	HBP1	located within a region known as the B-cell integration cluster (BIC)	ApoE^−/−^ mice	ox-LDL-treated human THP-1 macrophages	(+) Enhanced lipid uptake and enhanced ROS production	Silencing of miR-155 in ApoE^−/−^ mice by injecting antagomiR-155 decreased the lipid-laden macrophages and the formation of atherosclerotic plaques	[113]
**miR-216a**	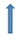	CSE	located on chromosome 2p16.1	-	THP-1 macrophages-derived foam cells	(+) Decreased ABCA1 expression and cholesterol efflux	Downregulation of CSE/H2S leads to an increase in cholesterol accumulation in foam cells	[114]
**miR-382-5p**	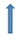	NFIA	located on chromosome 14q32.31	-	THP-1 macrophage-derived foam cells	(+) Reduces cholesterol efflux Increases lipid uptake	RP5-833A20.1/miR-382-5p/NFIA pathway regulates cholesterol homeostasis	[107]
**miR-486**	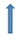	HAT1	located on chromosome 8p11.21	-	THP-1 macrophage-derived foam cells	(+) Reduces cholesterol efflux	HAT1 is capable of acetylating H4K5 and H4K12 and increasing ABCA1 expression	[115]
**miR-212**	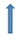	SirT1	located on chromosome 17p13.3	ApoE^−/−^ mice	THP-1 human macrophages treated with oxLDL	(+) Suppresses ABCA1 dependent cholesterol efflux	SIRT1 has capable of inducing LXR activity to increase ABCA1 expression in human macrophages	[116]
**miR-19a**	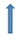	HBP-1	miR-19a is an important member of the miR-17–92 polycistronic gene cluster MiR-19a is abundant in the blood and tissues of patients with atherosclerotic coronary artery disease	ApoE^−/−^ mice	THP-1 derived macrophages RAW264.7 cells	(+) Increases lipid uptake of macrophages	HBP-1 participates in inhibiting the expression of the macrophage migration inhibitory factor (MIF) and lipid uptake by macrophages size of the atherosclerotic plaques in antagmiR-19a treated mice was reduced	[117]
**miR-497**	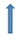	Apelin	Located on chromosome 17q13.1	-	Human THP-1 macrophages treated with oxLDL	(+) Decrease cholesterol efflux to apoA-I.	Apelin is an adipokine that is involved in the pathophysiology of cardiovascular diseases	[118]
**miR-20a/b**	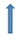	ABCA1	Mir 20a Located on chromosome 13q31.3 Mir 20b Located on chromosome Xq26.2	ApoE^−/−^ Mice	THP-1 and RAW 264.7 Macrophage-derived foam cells.	(+) Reducing cholesterol Efflux, impairs RCT in vivo	Both in in vitro studies and in ApoE^−/−^ mice treated with miR-20a/b, the hepatic expression of ABCA1, as well as reverse cholesterol transport, are decreased	[119]
**miR-758**	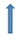	ABCA1	miR-758 was widely expressed in mouse tissues and particularly abundant in the brain, heart and aorta localized in an intergenic region within chromosome 14	Ldlr^−/−^ mouse	Mouse and human macrophage	(+) Cholesterol efflux to apoA1	effect of miR-758 on cholesterol efflux to ApoA1 was significantly attenuated after ABCA1 silencing decrease in peritoneal macrophages obtained from hypercholesterolemia *LDLR*^−/−^ mice	[120]
**LncRNA MALAT1**	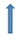	CD36, b-catenin	Located on chromosome 11q13.1	-	THP-1-derived macrophage	(+) Enhances lipid uptake	oxLDL induces MALAT1 transcription via the NF-kB pathway Knockdown of MALAT1 using siRNA transfection reduces CD36 expression and affects lipid uptake in macrophages	[121]
**LncRNA ENST00000602558.1**	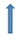	ABCG1		-	vascular smooth muscle cells (VSMCs)	(+) Reduce ABCG1-mediated cholesterol efflux to HDL	ENST00000602558.1 induces p65, which is a specific inhibitor of NF-kB and mediates a decrease in the expression of ABCG1	[122]
**LncRNA UCA1**	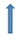	Mi R-206	downregulation of UCA1 inhibits oxidative stress and induces apoptosis of THP-1 cells, Located on chromosome 19p13.12	-	THP-1 cells	(+) Increase oxidative stress process In addition, CD36 levels	oxLDL greatly increases UCA1 expression, UCA1 ‘sponges’ miR-206 to exacerbate atherosclerosis	[123]

**Table 2:** Non-coding RNAs that attenuate foam cell formation/function.

Non-Coding RNA	Expression Type	Target Gene	Genetic Information (Human)	Experimental Model		Effect on Foam Cell Formation	Regulation in Lipid Metabolism	Reference
				In Vivo	In Vitro			
**miR-1275**	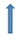	ApoC2	located on chromosome 6p21.31	-	THP-1 derived macrophages	(−) Inhibited the cellular uptake of Ox-LDL and lipid accumulation	ApoC2 most important cofactor for LPL lipolytic activity	[124]
**miR-202-3p**	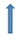	SCARB2, ABCG4, NCEH1I	located on chromosome 10q26.3	-	Human THP-1 macrophage-derived foam cell	(−) Attenuates lipid uptake Enhances cholesterol efflux	Reduces the expression of scavenger receptor SCARB2 Increases the expression of NCEH1 and ABCG4	[125]
**miR-181a**	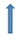	TLR4	located on chromosome 1q32.1	-	THP-1	(−) Attenuates lipid uptake and apoptosis and inflammation	Inhibits protein levels of CD36 (scavenger receptor class B)	[126]
**miR-135a**	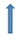	TLR4	located on chromosome 3p21.2	-	RAW264.7 and MOVAS cells	(−) Attenuates lipid uptake and inhibit oxidative stress and vascular inflammation	Inhibits CD36 expression	[127]
**miR-9**	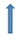	ACAT1	located on chromosome 1q22	-	Human THP-1 macrophage-derived foam cell	(−) Decrease the cholesterol ester formation	Reduces the levels of the ACAT1 protein (Acyl-coenzyme A:cholesterol acyltransferase )	[128]
**miR-21**	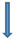	MKK3 MERTK	located on chromosome 17q23.1	Ldlr^−/−^ or miR21^−/−^ mice	Peritoneal macrophages from adult miR21^−/−^ mice	(−) Attenuated cholesterol efflux and promoting the lipid accumulation, Reduce apoptotic cell uptake	Knock down of miR-21 increases the expression of MKK3, promoting the induction of p38-CHOP and jNK signaling, which results in degradation of ABCG1, Decreases expression of MERTK; a key receptor that mediates the clearance of apoptotic cells	[129]
**miR-150**	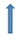	AdipoR2	located on chromosome 19q13.33	-	THP-1 macrophages	(−) Attenuates lipid uptake Increases cholesterol efflux	Decreases CD36, upregulates ABCA1 and ABCG1 via the PPARγ- and LXRα-dependent pathways.	[130]
**miR-133a**	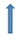	TR4 (testicular orphan nuclear receptor 4)	located on chromosome 18q11.2	-	RAW 264.7 macrophage cells	(−) Decreased oxLDL uptake In addition, lipid accumulation	Decreases expression of CD36	[131]
**miR-155**	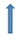	Tim-3, CEH (cholesterol ester hydrolase)	located on chromosome 21q21.3	-	THP-1	(−) Increases cholesterol efflux, Decreased lipid uptake	inhibits Tim-3 expression and increases the expression of CEH resulting in increased expression of ABCA1; inhibits the expression of SR-A,	[132]
**miR-155**	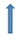	CARHSP1	located on chromosome 21q21.3	-	THP-1	(−) Decrease inflammation and lipid uptake	suppresses TNF-α production by directly targeting CARHSP1, which is required for TNF-α mRNA stabilization	[133]
**miR-98**	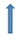	LOX-1 (Lectin-like ox-LDL scavenger receptor-1)	located on chromosome Xp11.22	ApoE^−/−^ mice	Macrophages were collected from C57BL/6 mice	(−) Decreases the lipid uptake and lipid accumulation	decreases LOX-1 expression	[134]
**miR-223**	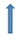	TLR4-NF-κB signaling pathway	located on chromosome Xq12	ApoE^−/−^ mouse, C57BL/6J wild-type mice(control)	Murine macrophage cell line RAW 264.7	(−) Increase cholesterol efflux to apoA-I, Decrease inflammation	activates the PI3K/AKT signaling pathway	[135]
**miR-23a**	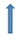	HSP90	located on chromosome 19p13.12	-	THP-1 macrophages	(−) Inhibit lipid accumulation	Decreased apoptosis of foam cells; decreases inflammation factors by inhibiting the activation of NF-κB pathways	[136]
**miR-146b-5p**	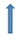	TRAF6	located on chromosome 10q24.32	-	THP-1 human monocytic and the HEK293T human embryonic kidney cell line	(−) Decrease lipid uptake	miR-146-5p inhibitor increases TRAF6-mediated activation of NF-κB (p65) and increases inflammatory response	[137]
**miR-221-3p**	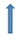	ADAM22	located on chromosome Xp11.3	-	RAW264.7	(−) Decrease lipid uptake	Decreases CD36; decrease oxidative stress and apoptosis	[138]
**miR-16**	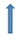	PDCD4 (Programmed cell death 4)	located on chromosome 13q14	ApoE^−/−^ mice	macrophage-derived foam cell (RAW264.7)	(−)	Suppresses the activation of inflammatory macrophages and decreases release of inflammatory cytokines via the MAPK and NF-κB pathways	[139]
**miR-34a**	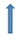	HDAC1 (histone deacetylase 1)	located on chromosome 1p36.22	Male C57BL/6J and ApoE^−/−^ mice	Human THP-1 macrophages	(−) Prevents the accumulation of triglyceride and total and free cholesterol	Hyperhomocysteinemia (HHcy) accelerates atherogenesis via decreased expression of miR-34a	[140]
**Let-7g**	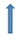	NF-κB complex MEKK1	located on chromosome 3p21.2	ApoE^−/−^ mouse, IKKαf/f:MLysCre/apoE^−/−^ mice	Human THP-1 macrophage	(−) increase cholesterol efflux, Decrease intracellular lipid accumulation	up-regulates SREBF2, which is a critical regulator of cholesterol/lipid homeostasis, up-regulates ABCA1 via suppression of miR-33a, decreases p53-dependent apoptosis	[141]
**LncRNA CDKN2B-AS1**	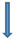	ADAM10	located on chromosome 9p21.3	ApoE^−/−^ Mice, C57BL/6J Mice	THP-1 macrophage-derived foam cells	(−) Increases Cholesterol Efflux, Decrease lipid accumulation	Inhibits inflammatory responses by suppressing the transcription of ADAM10	[142]
**LncRNA DYNLRB2-2**	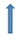	miR-298	located on chromosome 16q23.2	-	THP-1 macrophage-derived foam cells	(−) Impairing oxLDL uptake, increases cholesterol efflux	Induces autophagy by activating the LKB1/AMPK/mTOR signaling pathway via the miR-298/SIRT3 axis; decreases the expression of TLR2 and enhances the expression of ABCA1	[143]
